# Supplementary material for: Single-Molecule Mixture: A Concept in Polymer Science
Source: Int J Mol Sci. 2024 Jul 10;25(14):7571. doi: 10.3390/ijms25147571 (PMC11277297; doi:10.3390/ijms25147571)
Supplement: Supplementary file 1 [file ijms-25-07571-s001.zip › ijms-3091543-supplementary.pdf]

Supporting Information

Single Molecule Mixtures: A Concept in Polymer Science

Yu Tang

Figure S1. Schematic diagram showing single-molecule mixtures of substituted polymer system

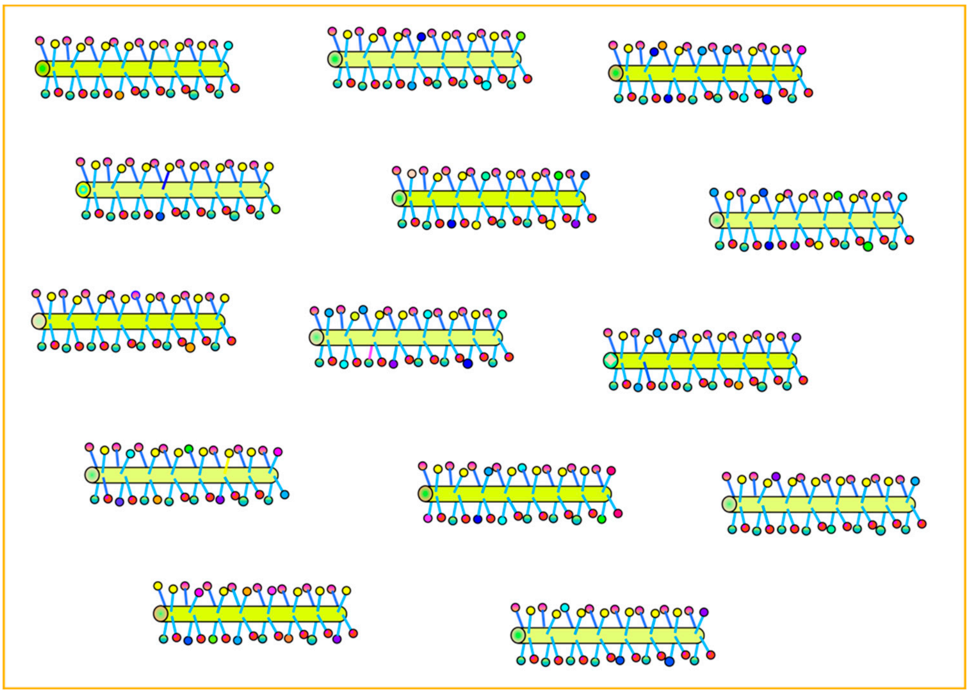

Table S1. Overall number of potential isomers of model system I.

n	**overall substitute rate（%）**	**Overall number of potential isomers (*r*)**	**lg*r***
0	0	1	0
1	0.1	1000	3
2	0.2	4.995×10^{5}	5.70
3	0.3	1.66×10^{8}	8.22
4	0.4	4.14×10^{10}	10.6
5	0.5	8.25×10^{12}	12.9
6	0.6	1.37×10^{15}	15.1
7	0.7	1.94×10^{17}	17.3
8	0.8	2.41×10^{19}	19.4
9	0.9	2.66×10^{21}	21.4
10	1.0	2.63×10^{23}	23.4
11	1.1	2.37×10^{25}	25.4
12	1.2	1.95×10^{27}	27.3
13	1.3	1.48×10^{29}	29.2
14	1.4	1.05×10^{31}	31.0
15	1.5	6.88×10^{32}	32.8
16	1.6	4.24×10^{34}	34.6
17	1.7	2.45×10^{36}	36.4
18	1.8	1.34×10^{38}	38.1
19	1.9	6.92×10^{39}	39.8
20	2.0	3.39×10^{41}	41.5
21	2.1	1.58×10^{43}	43.2
22	2.2	7.05×10^{44}	44.8
23	2.3	3.00×10^{46}	46.5
24	2.4	1.22×10^{48}	48.1
25	2.5	4.76×10^{49}	49.7

Table S2. Overall number of potential isomers of model system II.

n	**overall substitute rate（%）**	**Overall number of potential isomers (*r*)**	**lg*r***
0	0	1	0
1	1	1000	3
2	2	4.95×10^{5}	5.69
3	3	1.62×10^{8}	8.21
4	4	3.92×10^{10}	10.6
5	5	7.53×10^{12}	12.9
6	6	1.19×10^{15}	15.1
7	7	1.60×10^{17}	17.2
8	8	1.86×10^{19}	19.3
9	9	1.90×10^{21}	21.3
10	10	1.73×10^{23}	23.2
11	11	1.41×10^{25}	25.1
12	12	1.05×10^{27}	27.0
13	13	7.11×10^{28}	28.9
14	14	4.42×10^{30}	30.6
15	15	2.53×10^{32}	32.4
16	16	1.35×10^{34}	34.1
17	17	6.65×10^{35}	35.8
18	18	3.07×10^{37}	37.5
19	19	1.32×10^{39}	39.1
20	20	5.36×10^{40}	40.7
21	21	2.04×10^{42}	42.3
22	22	7.33×10^{43}	43.9
23	23	2.49×10^{45}	45.4
24	24	7.98×10^{46}	46.9
25	25	2.43×10^{48}	48.4

Figure S2. A Possible Synthetic Route Toward the 24 mer of *O*-propyl substituted D-mannitol model system.

A.

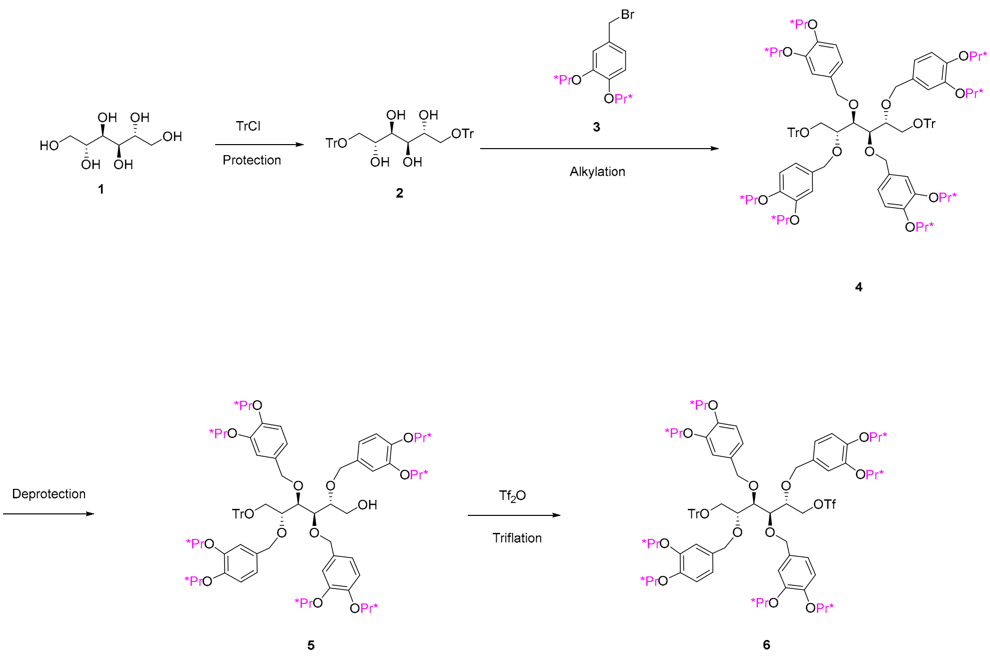

B.

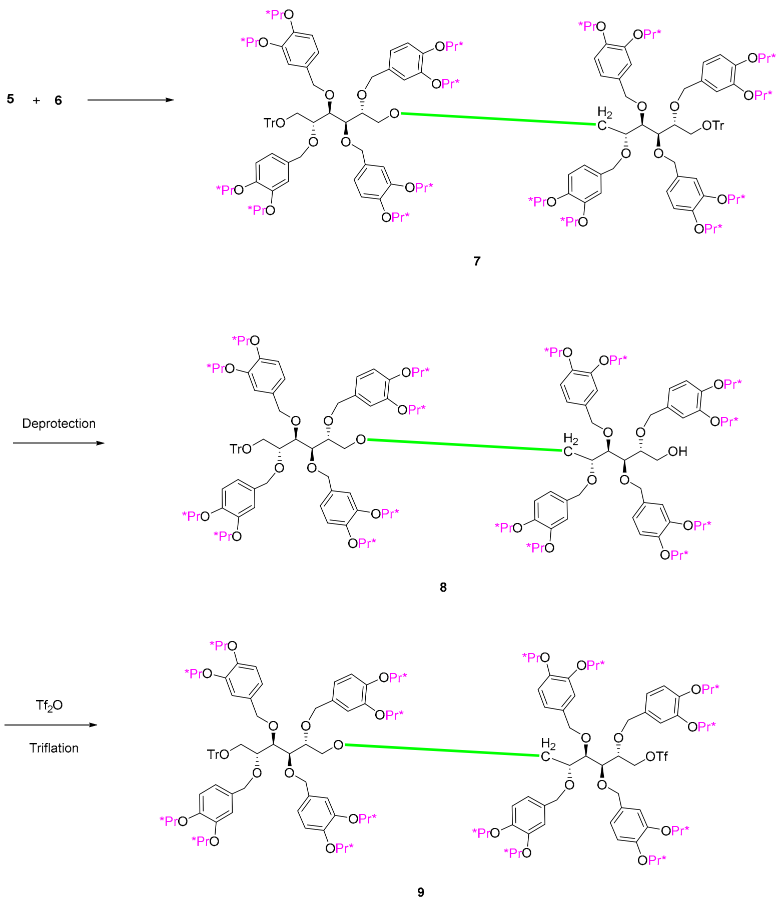

C.

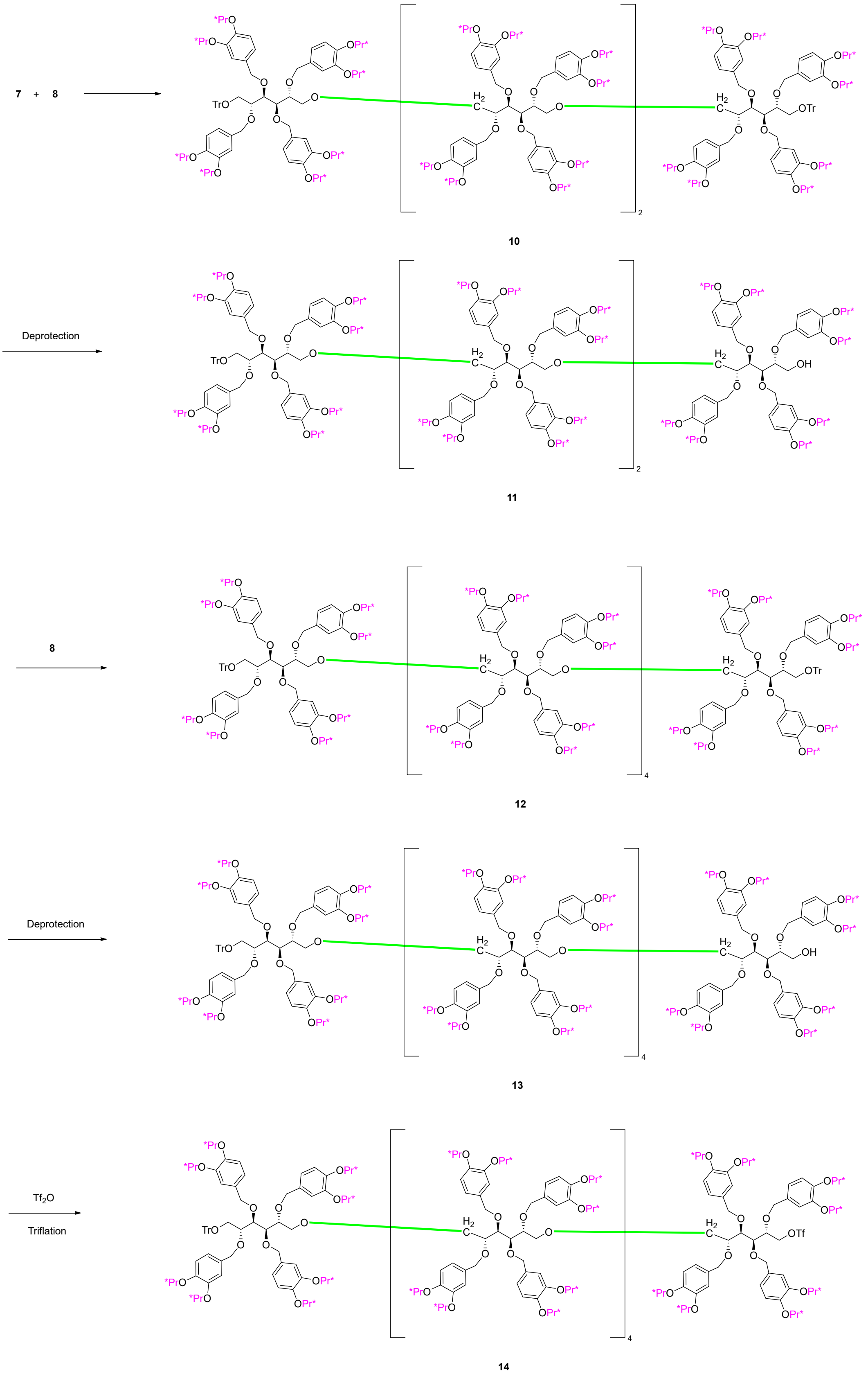

7 + 8
10
Deprotection
11
8
12
Deprotection
13
Tf_2O
Triflation
14

D.

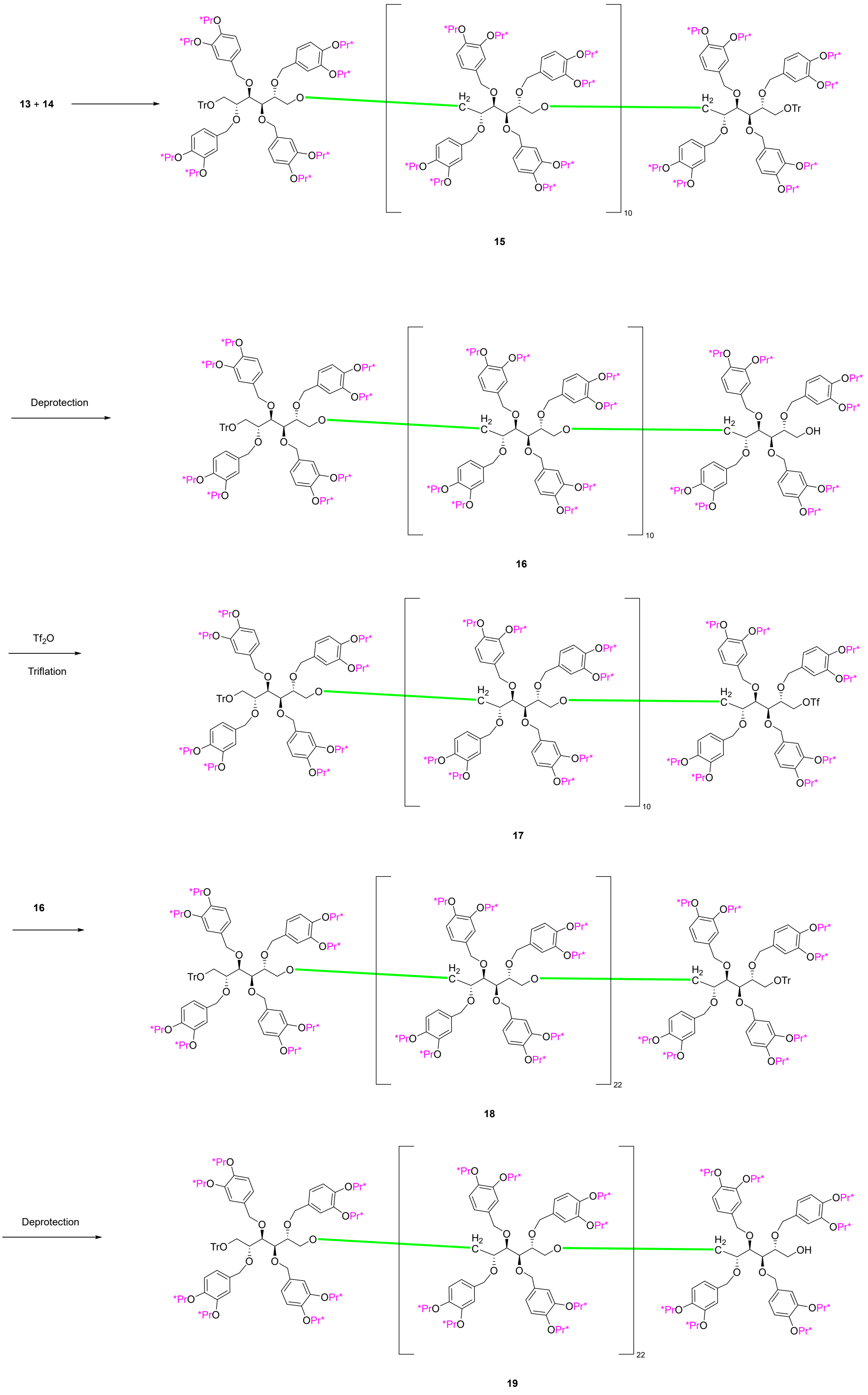
13 + 14
15
Deprotection
16
Tf2O
Triflation
17
16
18
Deprotection
19
TrO
OTr
OH
OTf
*PrO
OPr*
H2
10
22

A synthetic route of the single molecule mixture state 24-mer is illustrated in figure S2. In this route, equal mixtures of substituted benzyl bromide **3** are used, which are expected to have essentially the same reactivity and polarity. Thus, the synthetic and purification procedure are expected to be similar to synthesizing pure single-isomer products[1].

References

1. Y. Huang, Novel Crosslinking Reagents, Macromolecules, Therapeutic Conjugates, And Synthetic Methods Thereof. WO2013012961A2, 2013.1.24
